# Environmental stress alters genetic regulation of novelty seeking in vervet monkeys

**DOI:** 10.1111/j.1601-183X.2011.00707.x

**Published:** 2011-08

**Authors:** L A Fairbanks, J N Bailey, S E Breidenthal, M L Laudenslager, J R Kaplan, M J Jorgensen

**Affiliations:** 1 Department of Psychiatry and Biobehavioral Sciences, Semel Institute, University of CaliforniaLos Angeles (UCLA), Los Angeles, CA; 2 Department of Psychiatry, University of Colorado Anschutz Medical CampusDenver, CO; 3 Department of Pathology, Section on Comparative Medicine, Wake Forest University School of MedicineWinston-Salem, NC, USA

**Keywords:** Bivariate genetic correlation, environment, heritability, novelty seeking, stress, vervet monkey

## Abstract

Considerable attention has been paid to identifying genetic influences and gene–environment interactions that increase vulnerability to environmental stressors, with promising but inconsistent results. A nonhuman primate model is presented here that allows assessment of genetic influences in response to a stressful life event for a behavioural trait with relevance for psychopathology. Genetic and environmental influences on free-choice novelty seeking behaviour were assessed in a pedigreed colony of vervet monkeys before and after relocation from a low stress to a higher stress environment. Heritability of novelty seeking scores, and genetic correlations within and between environments were conducted using variance components analysis. The results showed that novelty seeking was markedly inhibited in the higher stress environment, with effects persisting across a 2-year period for adults but not for juveniles. There were significant genetic contributions to novelty seeking scores in each year (*h*^2^ = 0.35–0.43), with high genetic correlations within each environment (rhoG > 0.80) and a lower genetic correlation (rhoG = 0.35, non-significant) between environments. There were also significant genetic contributions to individual change scores from before to after the move (*h*^2^ = 0.48). These results indicate that genetic regulation of novelty seeking was modified by the level of environmental stress, and they support a role for gene–environment interactions in a behavioural trait with relevance for mental health.

Chronic and acute stressful life events are known to increase the risk for psychopathology in human populations (Hammen 2005; Kessler 1997), but not all individuals exposed to the same stressors will develop similar symptoms (Caspi *et al.* 2010; Rutter 2009; Wermter *et al.* 2010). Rodent models of chronic mild stress (CMS) have verified the link between persistent stress and behavioural, hormonal and neurochemical symptoms of pathology. CMS in rats and mice has been shown to produce behavioural changes in sucrose preference, learned helplessness and reduced exploratory behaviour that are consistent with depressive symptoms, and that are reversed by antidepressant medication (Wilner 2005). Research comparing different rat and mouse strains showed that CMS reduced the amount of time subjects spent in the centre of an open field or in the light area of a dark/light box in some strains, but not in others (Kirshenbaum *et al.* 2011; Mineur *et al.* 2006; Wu & Wang 2010), suggesting that there are genetic influences on the effects of CMS on exploratory behaviour.

In this report, we take advantage of a major environmental change for a pedigreed nonhuman primate colony to assess genetic influences on a measure of exploratory behaviour within and across environments differing in the level of stress. In early 2008, the Vervet Research Colony (VRC) was moved across country to a new facility to expand access to the colony for life span and metabolic research. The change involved the acute stress of the move, as well as an ongoing increase in the level of handling and group disturbances in the new location. A significant increase in hair cortisol from before to 6 months after the move provided independent evidence that the two locations differed in level of environmental stress (Fairbanks *et al.* in press). The relocation provided the opportunity to assess behaviour, heritability, and genetic correlations of novelty seeking within and across environments.

Novelty seeking is a behavioural trait that has been associated positively with vulnerability for substance abuse, and negatively with risk for depression and anxiety in humans and animal models (Cloninger *et al.* 1993; Pawlak *et al.* 2008). Prior results at the VRC have shown that the tendency to approach novel objects in a free-choice novelty test is influenced by genetic factors (Bailey *et al.* 2007). We have also shown that high levels of cortisol in hair are associated with suppressed novelty seeking for adult females in the colony (Laudenslager *et al.* 2011). The extended multigenerational pedigree at the VRC allows assessment of the heritability and genetic correlations of novelty seeking behaviour within and between environments differing in level of environmental stressors, and the heritability of individual response to a stressful life change. A high genetic correlation within environments coupled with a low genetic correlation between environments would support the existence of gene–environment interactions (Rainwater *et al.* 2010), and specifically, the moderation of genetic effects by the level of environmental stress (Caspi *et al.* 2010; Rutter 2009).

## Materials and methods

### Subjects

Subjects include 503 vervet monkeys (*Chlorocebus aethiops sabaeus*) (age 1–24 years) living in 16 multigenerational, matrilineal social groups in the VRC between 2006 and 2009. All subjects were born at the original VRC facility and raised in relatively stable social groups that were managed to reflect the natural social composition of vervet groups in the wild. Current VRC colony members are descendants of 57 wild caught founders (29 females and 28 males) captured in St. Kitts and Nevis, West Indies, and introduced into the colony between 1975 and 1983. The colony management practice of maintaining females in stable matrilines and transferring males between groups has produced one large, complex, extended pedigree spanning eight generations.

### Low and higher stress environments

The original VRC facility, established in 1975, was bounded by a high, opaque fence in a quiet park-like setting on the grounds of the Veterans Administration Greater Los Angeles Healthcare System, Sepulveda campus. The monkeys were housed in 16 outdoor enclosures varying in size from 30 to 117 m^2^ of ground area (mean = 61 m^2^), each with its own attached indoor shelter. The outdoor corrals had one or two large platforms and multiple perches, climbing structures and enrichment devices. Care was provided by a small and consistent staff of animal technicians. There were no changes in adult male group membership in 2006 and 2007. The animals were captured and anesthetized three times during the 2-year period, for an annual veterinary examination and a magnetic resonance imaging (MRI) assessment. The only other group disturbances in the 2 years prior to the move were for behavioural tests and infrequent clinical interventions.

In January–February 2008, the entire colony was moved across country by ground transport to a newly constructed facility at the Wake Forest University Primate Center. The 16 social groups were housed in 2 new buildings, with 8 enclosures per building. Similarities between the old and new facilities included: (1) a large outdoor area (89 m^2^) for each group with a platform, shelves for sitting, and enrichment devices that were similar in design and material to those in the pre-move environment; and (2) adult female and immature monkeys remained in the same social groups after the move and there were no significant changes in the matrilineal dominance hierarchies. Eleven of the 16 groups had new breeding adult males introduced in the new environment in January–February 2008, a procedure that has been used throughout the history of the colony to prevent inbreeding and mimic the natural processes of male emigration and immigration. M.J.J. served as scientific manager of the colony at both locations.

Environmental changes included the acute stress of the cross-country move, followed by a 10-week quarantine period when the entire colony was captured five times at 2-week intervals for TB testing. Ongoing environmental differences included: (1) more than a threefold increase in handling and group disturbances for veterinary examinations, sample collection and cognitive/behavioural testing; (2) an increase in the number and unpredictability of individuals involved in animal handling and care; (3) exposure to other groups, increased noise levels and potential sleep disruption in the indoor areas; (4) exposure to colder winter temperatures and confinement to the indoor area when the temperature fell below 35°F; and (5) multiple changes in diet composition and palatability.

Both facilities were fully accredited by the American Association for the Accreditation of Laboratory Animal Care (AALAC), and the colony was managed in accordance with the Guide for Care and Use of Laboratory Animals (1996) and The Psychological Well-Being of Nonhuman Primates (1998) before and after the move. All relevant procedures were approved by the Institutional Animal Care and Use committees of UCLA, the Department of Veterans Affairs, and Wake Forest University Health Sciences.

### Home Group Novelty test

The Home Group Novelty test is a standardized procedure to measure free-choice novelty seeking in the home enclosure. During the tests presented here, the door to the indoor area was closed, so all group members were present in the outdoor area. A novel and potentially threatening object was placed in an open container and positioned outside the chain link fence of the home enclosure, within reach of the animals and away from any of the preferred sitting or resting places. The identity of each animal observed within 1 m of the novel object was coded for each minute in a 30-min test session. A team of two to three observers familiar with identifying individual monkeys made a consensus determination of who was within 1 m. The area within 1 m of the object only occupied a small portion of the home enclosure, and it required voluntary choice on the part of the monkeys to approach the object.

The Home Group Novelty test was repeated once per year between 2006 and 2009, with predator-like objects as the novel stimuli (2006, cloth snake; 2007, plastic tarantula; 2008, small plastic alligator; 2009, cloth snake). Groups were tested in the summer or fall (June–October) in each of the four test years. The first post-move tests were conducted in June 2008, 4–5 months after relocation to the new environment. The second post-move tests were conducted in October 2009. Features of the outdoor areas and placement of the novel objects did not differ between the two environments. L.A.F. and M.J.J. managed the tests and recorded the data at both locations, and the primary observers in the post-move environment were trained at the pre-move facility.

### Novelty seeking scores and analysis

The latency to approach was determined by the first minute an animal was recorded within 1 m of the novel stimulus, and time near was measured by the number of 1-min intervals the animal was observed within 1 m. Animals which approached within 1 m in the first minute were given a latency score of 0 and those who never approached were assigned a latency score of 30. A composite score combining latency and time near was computed for each year [novelty seeking score = (30 − latency) + time near], with high scores indicating a quicker approach and greater interest in the novel object. Animals that never approached within 1 m had a score of 0.

To accommodate changes in the subject pool and subject age across the 4-year period, the effect of year and environment on novelty seeking scores were evaluated using a mixed model approach. The number of subjects evaluated per year was as follows: 2006, *n* = 408; 2007, *n* = 404; 2008, *n* = 406; 2009, *n* = 381, with 290 subjects measured in all 4 years. Age was categorized into five groupings based on maturational stage and behavioural change: juvenile (1–3 years), adolescent (4 years), young adult (5 years), middle adult (6–12 years) and older adult (13–24 years). Yearlings born in the post-move environment were not included as subjects. Pre-screening of the data indicated that there were no significant main effects or interactions between novelty seeking score and subject sex, dominance rank, or the addition of new adult males to the group in the prior 6 months. The final model included novelty seeking score as the dependent variable, animal identity as the subject variable and age, site, test year nested within site, and age × site interaction as fixed factors.

Standardized residuals, controlling age group, were computed for each test for the bivariate genetics analyses. The pre-move mean and post-move mean novelty seeking scores were computed from the average of the two standardized residual scores within each site. The pre–post change score was the difference of the pre-move and post-move mean scores.

### The VRC pedigree and statistical genetics analyses

The VRC pedigree was constructed using a panel of 14 highly polymorphic microsatellite markers to determine paternity and verify maternity (Newman *et al.* 2002). For the 503 subjects that contributed data to one or more years of this study, all had genetically verified mothers and 485 had genetically verified fathers. The remaining 18 subjects were assigned unique dummy-coded fathers. In the vervet polygamous mating system, the highest ranking male typically fathers more than half of the offspring. This mating pattern and the movement of adult males between groups at 3–4-year intervals resulted in a large number of half siblings. The sample of 503 phenotyped individuals included 377 mother-offspring, 128 father-offspring, and 104 grandparent-grandoffspring dyads, 64 full siblings, 437 maternal half siblings, 1421 paternal half siblings, and thousands of full or half avuncular and cousin dyads. Most of the phenotyped subjects (79%) had one or more maternal sibling in the dataset.

Heritability and genetic correlations were estimated by variance component models using SOLAR (Sequential Oligogenic Linkage Analysis Routines, version 4.1.5), a computer package designed for variance component and linkage analysis in pedigrees of arbitrary size and complexity (Almasy & Blangero 1998). The variance component analysis included an additive genetic component (matrix of genetic relationships among all subject pairs times the proportion of phenotypic variance attributable to genetic variation), plus the shared environmental effects (matrix of shared environmental variables times the proportion of variation attributable to those shared environmental effects), plus a term for the unique environment variation and error. Genetic contributions to the phenotypic covariance were estimated using relatedness information from the full pedigree. In this analysis, we measured the effects of shared maternal environments (*c*^2^) by incorporating a matrix identifying individuals that were raised by the same mother. Final models were the most parsimonious and included covariates and shared maternal effects only if their contribution to the variance met a threshold value of *P*≤ 0.05 using log likelihood ratio tests. Values of *P*≤ 0.05 were considered significant.

Bivariate analysis was performed to estimate the genetic correlation between novelty seeking scores within and between environments, using SOLAR. For this analysis, the phenotypic variance–covariance matrix is partitioned into the additive-genetic and environmental variance–covariance matrices using the full pedigree information. The additive genetic correlation (rhoG) estimates the additive effects of shared genes on the covariance of novelty seeking score for the 2 years within each environment, and for the mean scores between environments. A genetic correlation significantly different from zero indicates that the two measures share susceptibility genes. A genetic correlation of one indicates that the same genes are controlling individual differences in novelty seeking behaviour across tests (Mahaney *et al.* 1999; Martin *et al.* 2010).

## Results

### Novelty seeking by site: effects of environmental change

The descriptive statistics for the latency to approach and time near the novel objects, the correlations between the two measures, and the composite novelty seeking scores for each year are shown in Table 1. The proportion of the variance in the composite score that was accounted for by each component was between 0.83 and 0.96 for latency and between 0.55 and 0.65 for time near.

**Table 1:** Descriptive statistics for novelty seeking measures by year

		Latency		Time near		Latency × time near	Novelty seeking score	
Year	*N*	Mean (SD)	Min-Max	Mean (SD)	Min-Max	*R*	Mean (SD)	Min-Max
Pre-move 2006	408	5.0 (9.0)	0–30	7.6 (6.3)	0–26	−.49	32.7 (13.3)	0–56
Pre-move 2007	404	6.2 (10.2)	0–30	4.7 (4.6)	0–23	−.48	28.5 (13.1)	0–52
Post-move 2008	406	18.4 (13.7)	0–30	1.8 (3.4)	0–23	−.65	13.4 (16.1)	0–52
Post-move 2009	381	18.7 (13.6)	0–30	1.2 (3.3)	0–21	−.66	13.1 (16.0)	0–50

**Figure 1:**
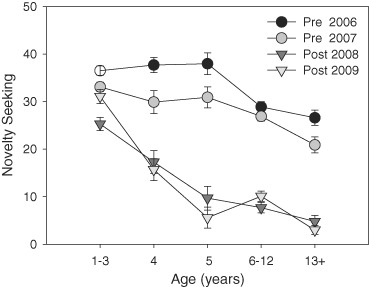
Novelty seeking within and between environments Mean (±SE) novelty seeking scores for vervet monkeys tested twice in a free-choice novelty test in the low-stress pre-move environment (pre 2006, pre 2007) and twice following the move to the higher stress environment (post 2008, post 2009) by age category.

Figure 1 shows the mean (±SE) novelty seeking scores by age category for the 4 test years. As the figure indicates, there were significant main effects of age (*F* = 388.3, df = 1/1593, *P* < 0.001) and site (*F* = 19.1, df = 1/1593, *P* < 0.001) and a significant interaction of age and site (*F* = 56.8, df = 1/1593, *P* < 0.001), with the effects of site considerably greater for adults than for juveniles aged 1–3 years. There was a significant effect of test year within site for the pre-move tests (*t* = 4.1, df = 807, *P* < 0.001) but not for the post-move tests [*t* = −1.4, df = 787, non-significant (ns)]. In both post-move years, the novelty seeking scores for adolescent and adult animals remained considerably below the pre-move levels.

While there were large mean differences in novelty seeking scores between sites, individual differences remained relatively consistent across years. The intra-class correlation coefficients were as follows: pre-move 2006–2007, ICC = 0.78, df = 363, *P* < 0.001; pre-move 2007 to post-move 2008, ICC = 0.55, df = 352, *P* < 0.001; post-move 2008–2009, ICC = 0.66, df = 362, *P* < 0.001; pre-move mean to post-move mean: ICC = 0.52, df = 289, *P* < 0.001).

### Heritability and genetic correlations of novelty seeking within and between environments

There were significant genetic contributions to variation in novelty seeking scores in each test year, with *h*^2^ ranging from 0.35 to 0.43 (Table 2). Age was a significant covariate in all 4 years, explaining 9% of the variance in the pre-move environment and 24–26% of the variance in post-move novelty seeking scores. The maternal effect did not contribute significantly to the variance in any of the 4 test years (*c*^2^ = 0.01 − 0.09, all ns).

**Table 2:** Heritability of novelty seeking scores

Novelty seeking	*n*	*h*^2^ (SE)	*P*	Variance × age (%)
Raw scores				
Pre-move 2006	408	0.35 (.09)	0.0000006	9
Pre-move 2007	404	0.38 (.09)	0.0000001	9
Post-move 2008	406	0.40 (.11)	0.0000001	24
Post-move 2009	381	0.43 (.11)	0.00000001	26
Age-adjusted summaries				
Pre-move mean	363	0.37 (.09)	0.0000005	
Post-move mean	380	0.58 (.10)	1.09e-13	
Pre-post change	290	0.48 (.13)	0.00003	

**Figure 2:**
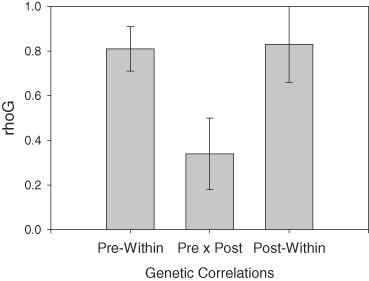
Genetic correlations within and between environments RhoG (+ SE) for two pre-move years (Pre-within), two post-move years (Post-within) and pre-move mean × post-move mean (Pre × Post).

Bivariate analyses within and between sites were conducted on the standardized residuals for each year, and for the pre-move and post-move means. The results, shown in Fig. 2, show high genetic correlations within site (rhog's > 0.80, *P* (from 0) <0.001), and a relatively low genetic correlation between sites (rhog = 0.34, *P* (from 0) = 0.06). Both of the within-site values were significantly greater than the between-site values (*t*'s > 3.0, *P* < 0.01). This suggests that there is a high degree of overlap in the genes influencing novelty seeking within each environment, with different genetic influences operating between the two environments. There was also a significant genetic component to the individual pre–post change scores (*h*^2^ = 0.48, Table 2), indicating a genetic influence on individual vulnerability to the increase in environmental stress.

## Discussion

The move from a low stress to a higher stress environment was associated with a dramatic decline in exploratory behaviour of vervet monkeys in a free-choice novelty test. The stressors in this study were not experimentally controlled, but they did involve changes in handling procedures, sleep environment, climate and diet that were largely unpredictable and uncontrollable from the animals' perspective. CMS protocols in rodents use a variety of similar stressors, including intermittent and unpredictable changes in the dark/light cycle, food availability, temperature or group membership. CMS studies produce behavioural changes that are consistent with depressive-like symptoms, including anhedonia, learned helplessness and reductions in exploratory behaviour in rats and mice (Kirshenbaum *et al.* 2011; Wilner 2005). The results presented here show a similar inhibition of voluntary exploratory behaviour in response to an increase in environmental stress for vervet monkeys.

An unanticipated finding from the present study was the relative resilience of juvenile monkeys to the environmental change. Juvenile novelty seeking was only slightly attenuated in the first year after the move, and did not differ from pre-move levels in the second year in the new environment. Adults, in contrast, showed a 70% mean reduction in novelty seeking scores that was maintained in the second year. Toth *et al.* (2008) also found age-dependent effects of CMS on depressive behaviour in rats, with adults showing the expected reduction in sucrose preference and exploratory behaviour, while juvenile behaviour was not affected. Neurochemical effects of the stress paradigm also differed markedly for adult and juvenile rats, suggesting that the behavioural resilience of the juveniles may be because of different effects of chronic stress on the developing brain.

While there were significant effects of environment on novelty seeking in this study, the results also verified the trait-like nature of novelty seeking, and showed the role of genetic influences within a consistent environment. Inhibition of behaviour and latency to explore novel objects have been shown to be heritable traits for infant and juvenile rhesus monkeys in novelty challenge tests (Rogers *et al.* 2008; Williamson *et al.* 2003). Prior research at the vervet colony showed that latency to approach a large novel object placed inside the home enclosures was heritable, with approximately 50% of the variance attributable to genetic factors (Bailey *et al.* 2007). The current study replicated the heritability of novelty seeking, using predator-like objects placed immediately outside the home enclosure. Comparison of novelty seeking scores from the initial test in 2003 with the tests conducted in 2006 and 2007 shows that response to the novel object tests remained relatively consistent over time within the pre-move environment (mean novelty seeking score: 2003 = 26.7; 2006 = 32.7; 2007 = 28.5), in contrast to the marked drop in scores in the higher stress post-move environment (2008 = 13.4; 2009 = 13.1).

Nonhuman primate models of the effects of environmental stress have used a variety of experimental paradigms, including group reorganization among adults, permanent or temporary separation of infants from their mothers, and variable work load for the mothers to assess the effects of stress on behaviour and development (Parker & Maestripieri 2010; Shively *et al.* 2005; Stevens *et al.* 2009; Suomi 1997). An important focus of this research has been on interactions between candidate gene polymorphisms and vulnerability to stressful life events during infancy (Kinally *et al.* 2010; Kraemer *et al.* 2008; Schwandt *et al.* 2010; Suomi 2006).

Complex behavioural disorders and conditions typically involve many genes with small effects, and replication of published effects of specific candidate genes on stress vulnerability and resilience in human studies has proven difficult (Risch *et al.* 2009). Epidemiological case–control studies have the challenge of consistently defining and measuring levels of environmental stress (Rutter 2009), and the problems of population stratification, gene–environment correlations and multiple testing which can lead to false-positive results (Dempfle *et al.* 2008; Jaffe & Price 2007; Lander & Schork 1994). In this report, the question of genetic influences on behavioural vulnerability to environmental stress was addressed with a common stressor and a within-subject longitudinal design using an extended pedigree. This design circumvents the problems of stratification and gene–environment correlations in case–control studies, and the difficulty of specifying levels of environmental stress in epidemiological research.

In other studies, genetic correlations have been used to identify genetic links between diagnosis and endophenotypes for childhood and adult psychiatric disorders, including attention deficit disorder, alcohol dependence and schizophrenia (Aukes *et al.* 2008; Distel *et al.* 2009; Grant *et al.* 2009; Ilott *et al.* 2010). Longitudinal designs have been used to show stability or change in genetic influences with age for childhood bipolar disorder and attention deficit disorder/hyperactivity disorder (Boomsma *et al.* 2006; Kuntsi *et al.* 2005). In the present study, longitudinal assessment of behaviour at two time-points within each environment allowed us to separate the effects of time from the effects of environmental change. The results indicated a high degree of consistency in the genetic influences on novelty seeking over time within the same environment, but considerably less overlap between environments that differed in apparent levels of stress. This result is consistent with a role for gene–environment interactions in genetic and environmental influences on novelty seeking behaviour. The low genetic correlation between environments suggests that different sets of genes are influencing individual differences in the low and higher stress environments.

Admittedly, there were limitations to the current study. The animals were subject to a diverse set of stressors in the new environment, and there was no control for changes over time unrelated to the move. Therefore, it is not possible in the current paradigm to ascribe the observed change in behaviour to any specific stressor. Research with mouse models of behavioural traits have showed that small changes in housing and test conditions can have significant and strain-dependent effects on outcome (Lewejohann *et al.* 2006; Mineur & Crusio 2009). In the current study, care was taken to use the same personnel and procedures within and across environments to minimize effects of Novelty test implementation on the responses of the monkeys. The genetic analyses showed strong consistency of genetics influences on novelty seeking within environments and a break in continuity between environments. These effects were derived from a large pedigreed population and likely reflect a robust response of socially housed monkeys to a stressful life change.

In summary, the results of this study were consistent with the hypothesis that an increase in environmental stress inhibits voluntary exploration and novelty seeking in vervet monkeys, with more pronounced effects on older than younger individuals. The results also showed a strong genetic contribution to behavioural vulnerability to stress, and provided evidence that genetic regulation of novelty seeking behaviour is modified by the level of environmental stress. This supports a role for gene–environment interactions in a behavioural trait with relevance for psychopathology, and underscores the difficulty in identifying genes that are consistently associated with complex behavioural traits across environments.
